# S100A7 as a potential diagnostic and prognostic biomarker of esophageal squamous cell carcinoma promotes M2 macrophage infiltration and angiogenesis

**DOI:** 10.1002/ctm2.459

**Published:** 2021-07-08

**Authors:** Zhiliang Lu, Sufei Zheng, Chengming Liu, Xinfeng Wang, Guochao Zhang, Feng Wang, Sihui Wang, Jianbing Huang, Shuangshuang Mao, Yuanyuan Lei, ZhanYu Wang, Nan Sun, Jie He

**Affiliations:** ^1^ Department of Thoracic Surgery State Key Laboratory of Molecular Oncology/National Cancer Center/National Clinical Research for Cancer/Cancer Hospital Chinese Academy of Medical Sciences and Peking Union Medical College Beijing China

**Keywords:** angiogenesis, biomarker, ESCC, S100A7, tumor‐associated macrophages

## Abstract

Dysregulated expression of S100A7 is found in several cancers and plays an important role in tumor progression; however, its carcinogenic role in esophageal squamous carcinoma (ESCC) is still poorly understood. Here, we identified that the levels of S100A7 were remarkably upregulated in 341 tumor tissues (*P* < .001) and 274 serum samples (*P* < .001) of ESCC patients compared with normal control. It was an independent prognostic factor (*P* = .026). Furthermore, a new diagnostic model for ESCC based on serum S100A7, SCC, and crfra21‐1 was established with area under curve (AUC) up to 0.863 (95% CI: 0.802‐0.925). Mechanically, we found upregulated S100A7 could promote cell migration and proliferation through intracellular binding to JAB1 and paracrine interaction with RAGE receptors and then activates the downstream signaling pathways. In addition, exocrine S100A7 could promote M2 macrophage infiltration and polarization by up‐regulating M2 macrophage associated proteins, and tumor angiogenesis by enhancing the activation of p‐ErK and p‐FAK pathways. Further animal experiments confirmed the role of S100A7 in promoting M2 macrophage infiltration and angiogenesis in ESCC. In conclusion, these findings highlighted the potential diagnostic and prognostic value of S100A7 in patients with ESCC. Meanwhile, our results reveal that S100A7 promotes tumor progression by activating oncogenic pathways and remodeling tumor microenvironment, which paving the way for the progress of S100A7 as a therapeutic target for cancer treatment.

## Graphical abstract

1. Elevated S100A7 in tumor tissues and serum samples of ESCC patients suggest a role as a biomarker.
2. A novel and promising diagnostic model for ESCC based on serum S100A7, SCC, and crfra21‐1 was established.
3. S100A7 promotes tumor progression by activating oncogenic pathways and remodeling tumor microenvironment.

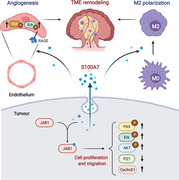

## 1 INTRODUCTION

Esophageal cancer, with an estimated 572 000 new cases per year worldwide, is the seventh most common cancer and the sixth leading cause of cancer death around the world.^1^ More than 80% of esophageal cancers are esophageal squamous cell carcinomas (ESCC).^2^, ^3^, ^4^ Despite advances in traditional therapeutics, targeted therapies, and immunotherapies, only 15‐25% of patients with ESCC survive for 5 years after diagnosis.^4^ It is therefore urgent to elucidate the molecular mechanisms of esophageal cancer development and to find effective diagnostic and prognostic markers.

The S100 superfamily of more than 20 calcium ion‐binding proteins is widely involved in multiple biological processes of various malignant tumors, including proliferation, migration, invasion, angiogenesis, immune escape, and cell differentiation.^5^ Dysregulated expression of S100 proteins is a common feature of carcinomas, and each type of carcinoma presenting a unique S100 protein signature.^6^, ^7^ Accordingly, several S100 proteins are important diagnostic markers and therapeutic targets for cancer treatment.

S100A7, also known as psoriasin, was first identified in the epidermal cells of patients with psoriasis in 1991 and was later determined to be a marker of human psoriasis lesions.^8^, ^9^, ^10^ In vivo, S100A7 exists as a bioactive homodimer called caltrophin and has several unique intracellular and extracellular functions. Under pathological conditions, elevated intracellular S100A7 expression is associated with enhanced proliferation and metastasis of tumor cells.^11^, ^12^, ^13^ Furthermore, once secreted, S100A7 may act as a mediator for interaction between tumor cells and the tumor microenvironment. Excreted S100A7 can bind to receptors like receptor for advanced glycation end‐product (RAGE) and Toll‐like receptor 4 (TLR4) to exert paracrine effects, thus promoting immune cell recruitment and vascular endothelial cell proliferation.^14^, ^15^, ^16^ S100A7 is upregulated in several types of malignancies including oral squamous cell carcinoma (OSCC), nonsmall cell lung cancer (NSCLC), breast cancer (BRCA), and skin cutaneous melanoma, leading to tumor growth, premetastatic niche formation, and metastasis.^13^, ^17^, ^18^ Although many studies have reported the biological functions and molecular mechanisms of S100 proteins in specific cancers, the role of S100A7 in ESCC remains poorly understood.

Here, we reveal the biological function and molecular mechanism of S100A7 in ESCC. In addition to promoting cancer cell progression, S100A7 promotes tumor‐associated macrophage infiltration and angiogenesis, thus supporting the development of the tumor microenvironment. We evaluate the clinical value of S100A7 as a diagnostic and prognostic marker, providing new ideas for the diagnosis and treatment of ESCC.

## 2 RESULTS

### 2.1 S100A7 is upregulated in ESCC and indicates poor prognosis

**FIGURE 1:**
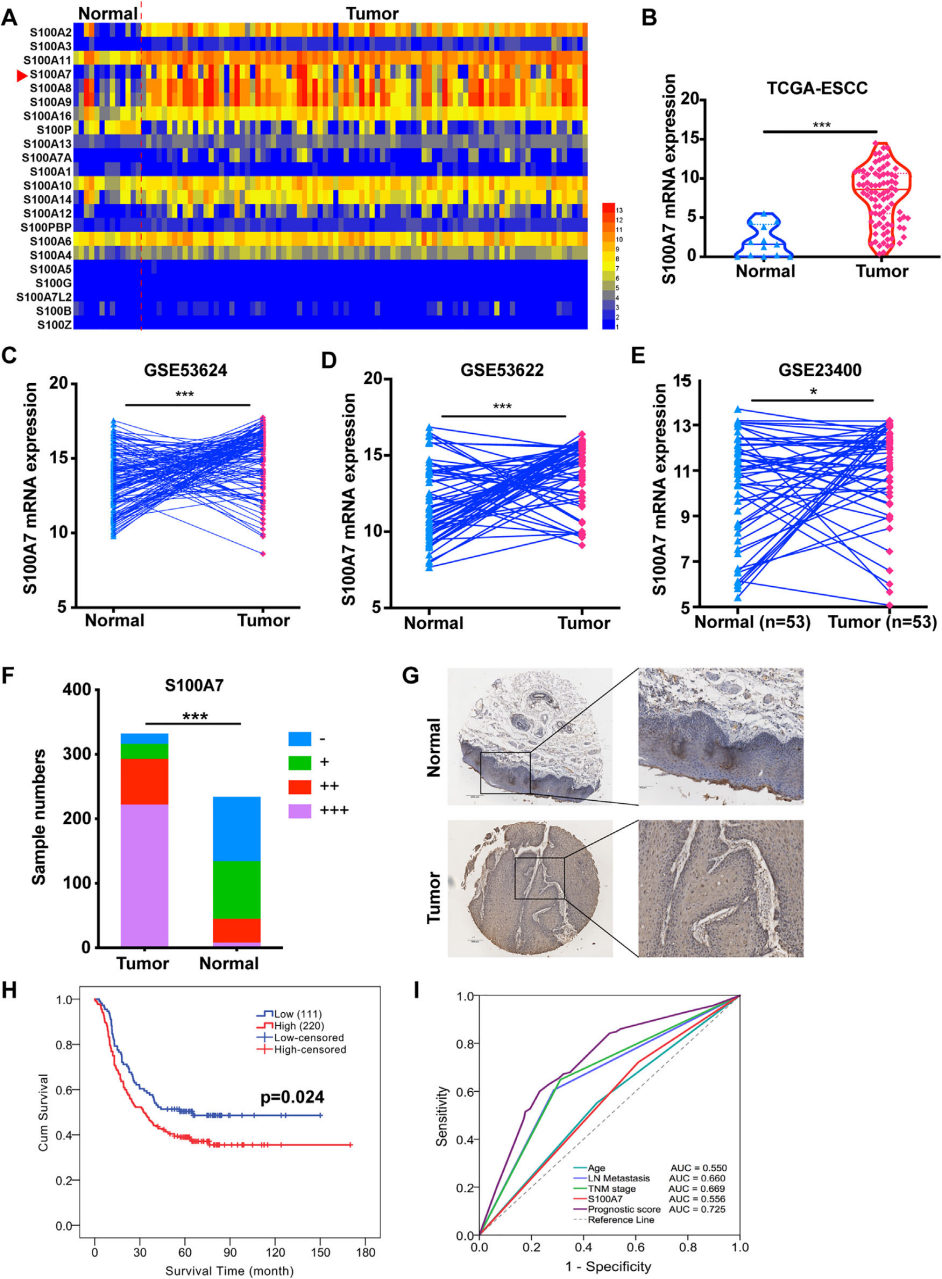
S100A7 is upregulated in ESCC and indicates poor prognosis. (A) Heat map of the mRNA expression level of S100 family proteins in the TCGA ESCC data set. (B) Violin diagram of S100A7 mRNA expression levels in ESCC and normal esophageal tissues in the TGCA database. (C‐E) Expression levels of S100A7 mRNA in three independent data sets (GSE53624, GSE53622, and GSE23400, respectively) of ESCC tissues and paired adjacent normal tissues. (F) Immunohistochemical analysis of the expression level of S100A7 protein in 341 ESCC tissues and 233 adjacent normal tissues. “‐”, negative; “+”, weak positive; “++”, medium positive; “+++”, strong positive. (G) Representative pictures of S100A7 immunodetection in ESCC tissues and adjacent esophageal tissues. (H) Survival curve of patients with ESCC with high and low S100A7 expression. (I) ROC curve of prognosis prediction for four independent prognostic indicators (age, lymph node metastasis, TNM stage, and S100A7 expression) in multi‐factor analysis. The prognostic score is determined by logistic regression analysis of the independent prognostic indicators. *P < .05, ***P < .001

We acquired transcriptome data from The Cancer Genome Atlas (TCGA) database and compared the mRNA expression levels of S100 family members between normal esophageal and ESCC tissues. Several S100 family members had abnormal mRNA expression in the ESCC tissues, including the well‐known S100A8, S100A9, and S100A2 proteins (Figure 1A). In addition, the less well‐known S100A7 protein was clearly upregulated in the ESCC tissues in comparison with the normal esophageal tissues (Figure 1B). The upregulation of S100A7 mRNA was subsequently verified in three independent RNA microarrays (GSE43624, GSE53622, and GSE23400), which comprised 119, 60, and 53 pairs of human ESCC and adjacent normal tissues, respectively^19^, ^20^ (Figure 1C‐E). Immunohistochemical analysis of 341 ESCC tissues and 233 adjacent normal tissue samples demonstrated that S100A7 protein levels were significantly increased in the ESCC tissues (Figure 1F,G, *P* < .001). The patients were assigned into two groups with high (immunohistochemical score ≥ 6) and low (immunohistochemical score < 6) S100A7 expression, respectively. There were no significant differences in age, gender, smoking status, or TNM staging between the two groups; however, the patients with high S100A7 expression had more advanced tumor differentiation than those with low S100A7 expression (*P* = .001, Table 1). Our study showed that the patients with high S100A7 expression also had shorter survival than those with low S100A7 expression by Kaplan‐Meier survival analysis (*P* = .024, Figure 1H). Meanwhile, we justified that S100A7 was an independent prognostic factor associated with poor prognosis by Multivariate Cox analysis (*P* = .026; odds ratio [OR] = 1.513, 95% CI: 1.094‐2.092, Table 2). Furthermore, prognosis prediction analysis showed that the addition of S100A7 expression as a prognostic factor increased the accuracy of prognosis assessment of patients with ESCC (Figure 1I). Collectively, the results showed that S100A7 was upregulated in ESCC tissues and was an independent prognostic factor.

**TABLE 1:** Correlation analysis between the expression level of S100A7 in ESCC tissues and clinicopathological characteristics

	S100A7		
	Low (*n* = 110)	High (*n* = 221)	*P* value
**Age**			
<60	53 (32.1%)	112 (67.9%)	0.727
≥60	57 (34.3%)	109 (65.7%)	
**Gender**			
Male	85 (33.2%)	171 (66.8%)	0.983
Female	25 (33.3%)	50 (66.7%)	
**Smoking**			
Nonsmoker	41 (35%)	76 (65%)	0.627
Smoker	69 (32.2%)	145 (67.8%)	
**Drinking**			
Nondrinker	46 (30.7%)	104 (69.3%)	0.414
Drinker	63 (35%)	117 (65%)	
**Family history**			
No	88 (32.2%)	185 (67.8%)	0.444
Yes	22 (37.9%)	36 (62.1%)	
**Location**			
Upper	18 (35.3%)	33 (64.7%)	0.325
Middle	55 (29.9%)	129 (70.1%)	
Lower	37 (33.6%)	59 (61.5%)	
**Differentiation**			
High	18 (22.8%)	61 (77.2%)	0.001*
Middle	57 (31.0%)	127 (69.0%)	
Low	35 (51.5%)	33 (48.5%)	
**T stage**			
T1	6 (42.9%)	8 (57.1%)	0.224
T2	11 (23.4%)	36 (76.6%)	
T3	74 (32.9%)	151 (67.1%)	
T4	19 (42.2%)	26 (57.8%)	
**Lymph node metastasis**			
Negative	61 (33.5%)	121 (66.5%)	0.907
Positive	49 (32.9%)	100 (67.1%)	
**TNM stage**			
I‐II	54 (31.6%)	117 (68.4%)	0.559
III‐IV	55 (34.8%)	103 (65.2%)	

* Indicates statistically significance.

**TABLE 2:** Survival analysis of S100A7 protein expression in ESCC tissues

				95% CI for OR	
	Univariate analysis	COX multivariate analysis	OR	Lower	Upper
Age	0.013	0.045*	1.316	0.987	1.751
Differentiation	0.002	0.139	1.577	01.126	2.212
Lymph node					
Metastasis	0.000	0.010*	1.681	1.136	2.481
TNM stage	0.000	0.007*	1.664	1.121	2.469
S100A7	0.024	0.026*	1.513	1.094	2.092

* Indicates statistically significance.

### 2.2 Serum S100A7 is a potential diagnostic biomarker for ESCC

**FIGURE 2:**
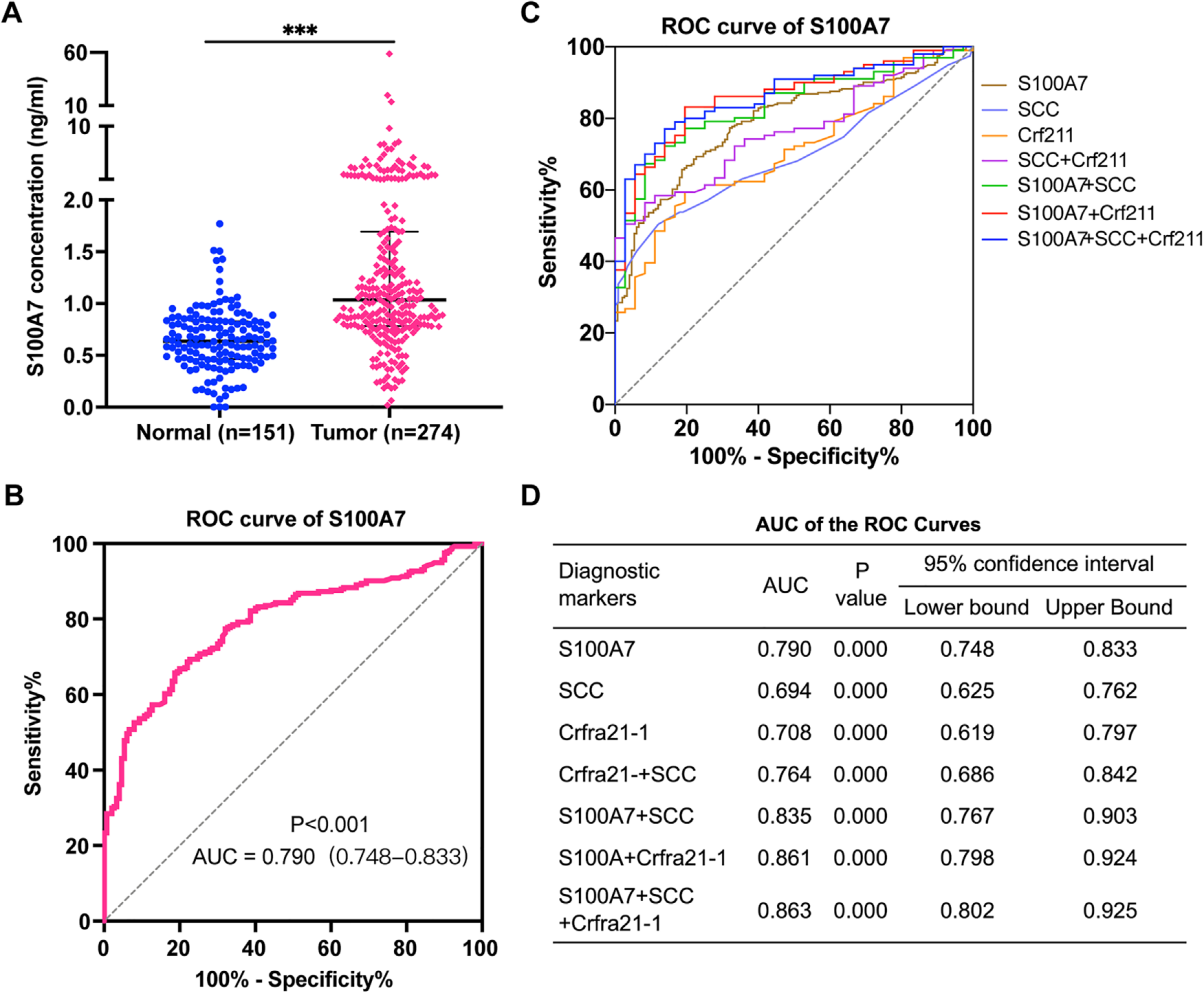
Serum S100A7 is a potential noninvasive diagnostic marker for ESCC. (A) ELISA analysis of the expression level of free S100A7 in serum of patients with ESCC and healthy controls. (B) ROC curve of serum S100A7‐based diagnosis of ESCC. (C) ROC curve of ESCC diagnosis based on serum S100A7 and the clinical markers SCC and crf211 as well as the combination of different markers. (D) The area under the ROC curve (AUC) for the diagnosis of ESCC with different combinations of diagnostic markers. ***P < .001

A previous study showed that S100A7 could be secreted outside the cell and detected in serum.^21^ ELISA analysis showed that the level of secreted S100A7 protein was significantly higher in serum samples from 234 patients with ESCC than in those from 127 healthy control individuals (*P* < .001, Figure 2A). And receiver operating characteristic (ROC) analysis demonstrated that the detection of S100A7 in serum had considerable diagnostic efficacy in patients with ESCC (Figure 2B). The area under the ROC curve (AUC) for diagnosis based on serum S100A7 was as high as 0.790 (95% CI: 0.748‐0.833, *P* < .001) with specificity up to 90.67% (95% CI: 84.94‐94.36%) when the sensitivity was 53.65% (95% CI: 47.74‐59.46%). Logistic regression analysis was used to integrate S100A7 with the widely used auxiliary diagnostic markers SCC and CRFRA21‐1. The inclusion of S100A7 as a diagnostic factor for ESCC increased the AUC from 0.764 (95% CI: 0.686‐0.842) for diagnosis based on SCC and crfra21‐1 to 0.863 (95% CI: 0.802‐0.925) for diagnosis based on S100A7, SCC, and crfra21‐1 (Figure 2C, D). Taken together, the results indicated that the serum S100A7 protein level has great potential as a noninvasive diagnostic marker for ESCC.

### 2.3 S100A7 promotes oncogenic signaling activation via binding with JAB1 and paracrine interaction

**FIGURE 3:**
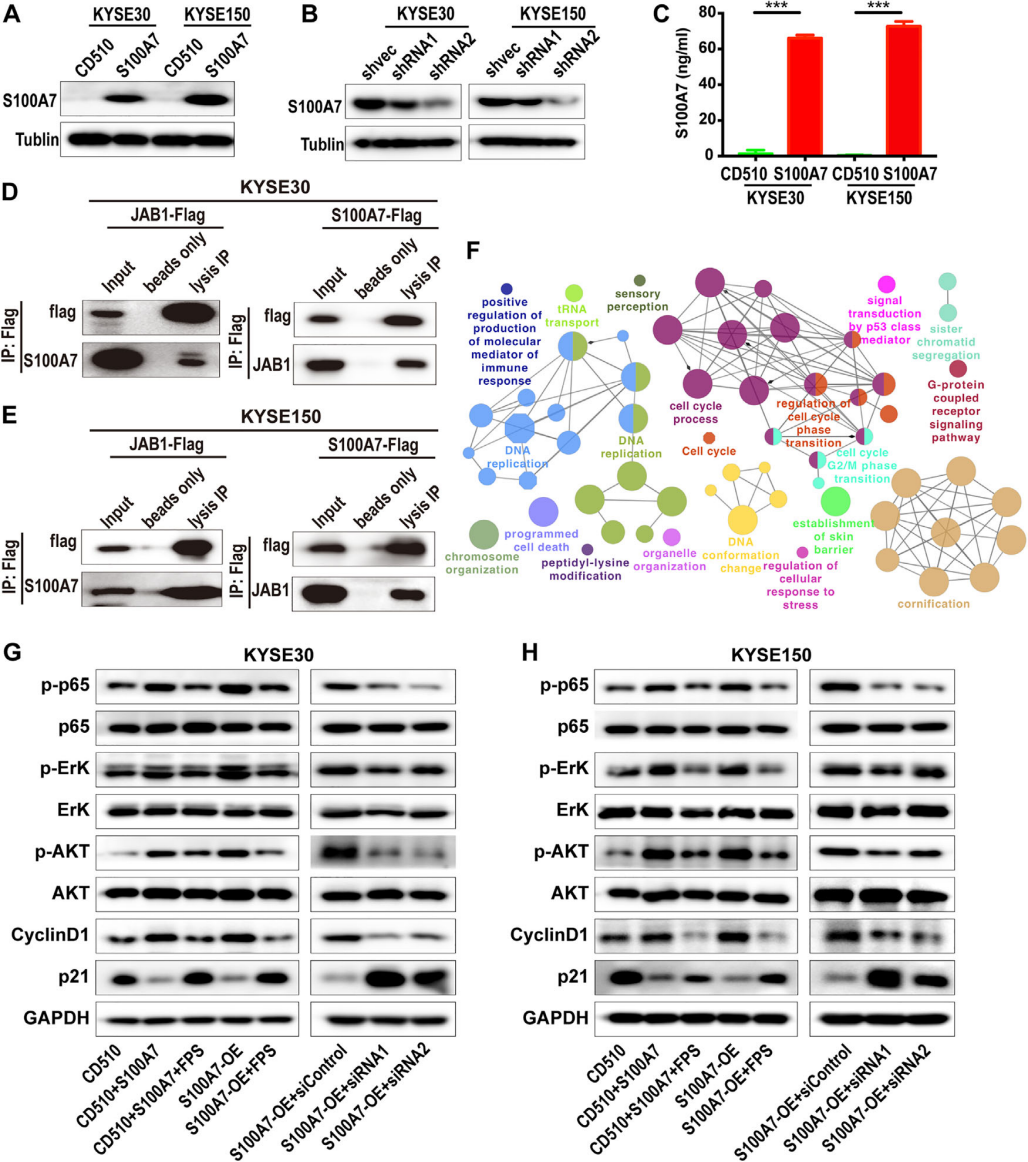
S100A7 binds with JAB1 and promotes activation of downstream oncogenic signaling. (A) The overexpression efficiency of S100A7 in KYSE‐30 and KYSE‐150 cells was shown by western blot. (B) Restorative silencing of S100A7 expression in S100A7‐overexpressing ESCC clones. (C) The concentration of secreted free S100A7 protein in culture supernatant of S100A7‐overexpressing and control ESCC clones. (D and E) Co‐immunoprecipitation analysis of the interaction of intracellular S100A7 and JAB1 in (D) KYSE‐30 and (E) KYSE‐150 cells. Beads only was used as a blank control. (F) Functionally grouped network analysis of genes differentially expressed between TCGA ESCC data sets with high and low S100A7 expression. KEGG pathways and gene ontology functional terms served as nodes linked on the basis of kappa scores (≥0.5). The label of the hub term in each group is presented. The larger the nodule, the more significant the enrichment. Different colors indicate its functional group. (G and H) Western blot analysis of JAB1 downstream signaling pathways. GAPDH was loading control. FPS and FPS‐ZM, RAGE‐specific inhibitors; S100A7, recombinant human S100A7 protein; S100A7‐OE, S100A7 overexpressed cells. ***P < .001

Previous studies reported that intracellular S100A7 can interact with C‐Jun activation domain‐binding protein‐1 (JAB1), whereas extracellular S100A7 might exert functions through the RAGE receptor.^14^, ^22^, ^23^ JAB1 was initially identified as a c‐Jun coactivator, and subsequently revealed to be the fifth component of the constitutive photomorphogenic‐9 signalosome (COPS5). Dysregulation of JAB1 is widespread in cancer and involved in affecting a series of cancer associated pathways by activating oncogenes and deactivating several tumor suppressors.^24^ To determine the role of S100A7 in the activation of ESCC signaling pathways, we constructed KYSE‐30 and KYSE‐150 cell lines that stably overexpressed S100A7 (Figure 3A). We then established additional KYSE‐30 and KYSE‐150 cell lines in which the S100A7 overexpression was restoratively silenced by siRNA (Figure 3B). The S100A7 overexpression increased the amount of secreted S100A7 protein in the culture supernatant to more than 60 ng/mL, which was much higher than the level in cultures of control cells without S100A7 overexpression (Figure 3C). Anti‐S100A7 coimmunoprecipitation assays and mass spectrometry analysis revealed that S100A7 was able to bind to the JAB1 protein, which was verified by western blot (Figure 3D,E). Kyoto Encyclopedia of Genes and Genomes (KEGG) pathway and gene ontology enrichment analysis of the transcriptome data of S100A7‐overexpressed cells and the TCGA ESCC cohort showed that genes that were differentially expressed between tumor cells with high and low S100A7 expression were enriched with functions related to tumor development and progression (Figures 3F and S1), which suggested that S100A7 plays a role in the activation of ESCC‐related signal pathways. Western blot analysis showed that S100A7 overexpression or exogenous treatment with S100A7 protein could promote activation of the AKT, ERK, and NFκB signaling pathways (Figure 3G,H). Those effects could be partly abrogated by S100A7 silencing or treatment with RAGE receptor inhibitors (Figure 3G,H). Taken together, the results suggested that S100A7 promotes activation of oncogenic signaling in ESCC cells via binding with JAB1 and autocrine interaction.

### 2.4 Upregulated S100A7 accelerates the proliferation of ESCC cells via apoptosis repression

**FIGURE 4:**
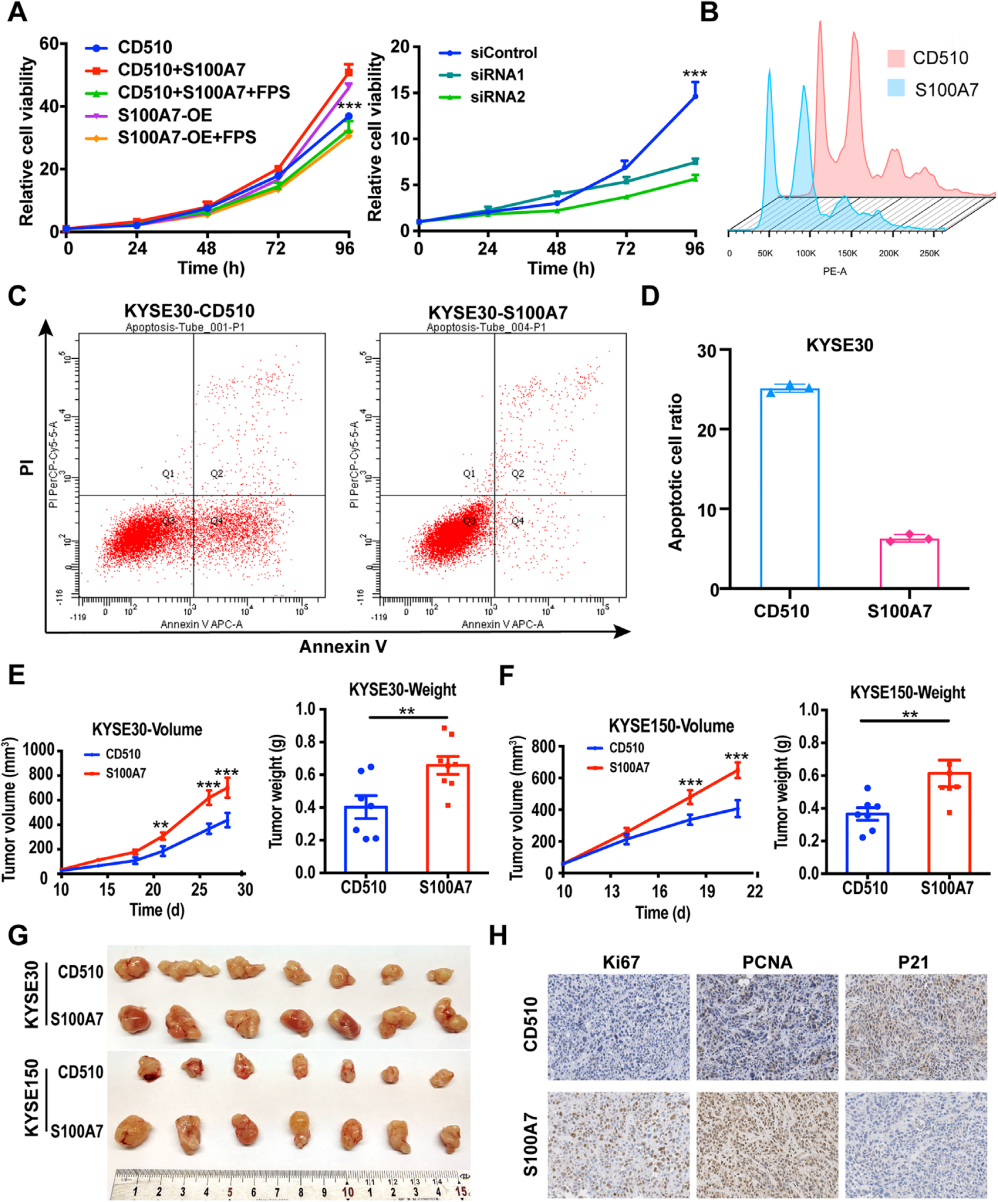
Upregulated S100A7 promotes cell proliferation in vitro and in vivo. (A) The left side shows the proliferation curves of S100A7‐overexpressing and control cells treated with or without recombinant human S100A7 protein or the RAGE‐specific inhibitor FPS‐ZM1. The right side shows the proliferation curves of restoratively S100A7‐silenced clones and control cells. (B) Flow cytometric analysis revealed that S100A7 had no effect on the cell cycle. (C) Representative results of flow cytometric apoptosis analysis. (D) Cisplatin‐induced apoptotic cell ratio of S100A7‐overexpressing and vector‐control cells. The growth curve and tumor nodule weight of xenograft tumors composed of the indicated (E) KYSE‐30 and (F) KYSE‐150 cell clones. (G) Images of excised xenograft tumors derived from vector‐control and S100A7‐overexpressing ESCC cells. (H) Immunohistochemistry analysis of the protein expression levels of Ki67, PCNA, and p21 in excised xenograft tumors from each indicated group. **P < .01, ***P < .001.

Because S100A7 appeared to be involved in cell proliferation‐related signaling pathways (Figures 3F and S1), we conducted CCK8 assays to determine its effect on the proliferation of ESCC cells. Consistent with the changes in signaling pathways, S100A7 overexpression or stimulation with exogenous S100A7 protein increased cell proliferation activity, whereas S100A7 knockdown or treatment with RAGE receptor inhibitor reduced proliferation activity (Figures 4A and S2A). Although the regulation of cell proliferation is complicated, cell cycle regulation and apoptosis are the most important and widely recognized pathways involved. Flow cytometry was applied to detect the cell cycle distribution of clonal populations. The results showed that S100A7 had no effect on the cell cycle distribution of KYSE‐30 or KYSE‐150 cells (Figures 4B and S2B); however, S100A7 overexpression inhibited cisplatin‐induced apoptosis of ESCC cells (Figures 4C,D and S2C, S2D). Furthermore, in subcutaneous xenograft experiments, S100A7‐overexpressing ESCC cells had increased tumor‐formation ability compared with ESCC cells expressing empty vector (Figure 4E‐G). Immunohistochemical analysis showed that the S100A7‐overexpressing xenograft tumors had higher Ki67 and proliferating cell nuclear antigen (PCNA) expression and lower P21 expression than the control tumors (Figure 4H). Overall, the results indicated thatS100A7 accelerates the proliferation of ESCC cells via apoptosis repression.

### 2.5 S100A7 promotes the metastatic capability of ESCC cells

**FIGURE 5:**
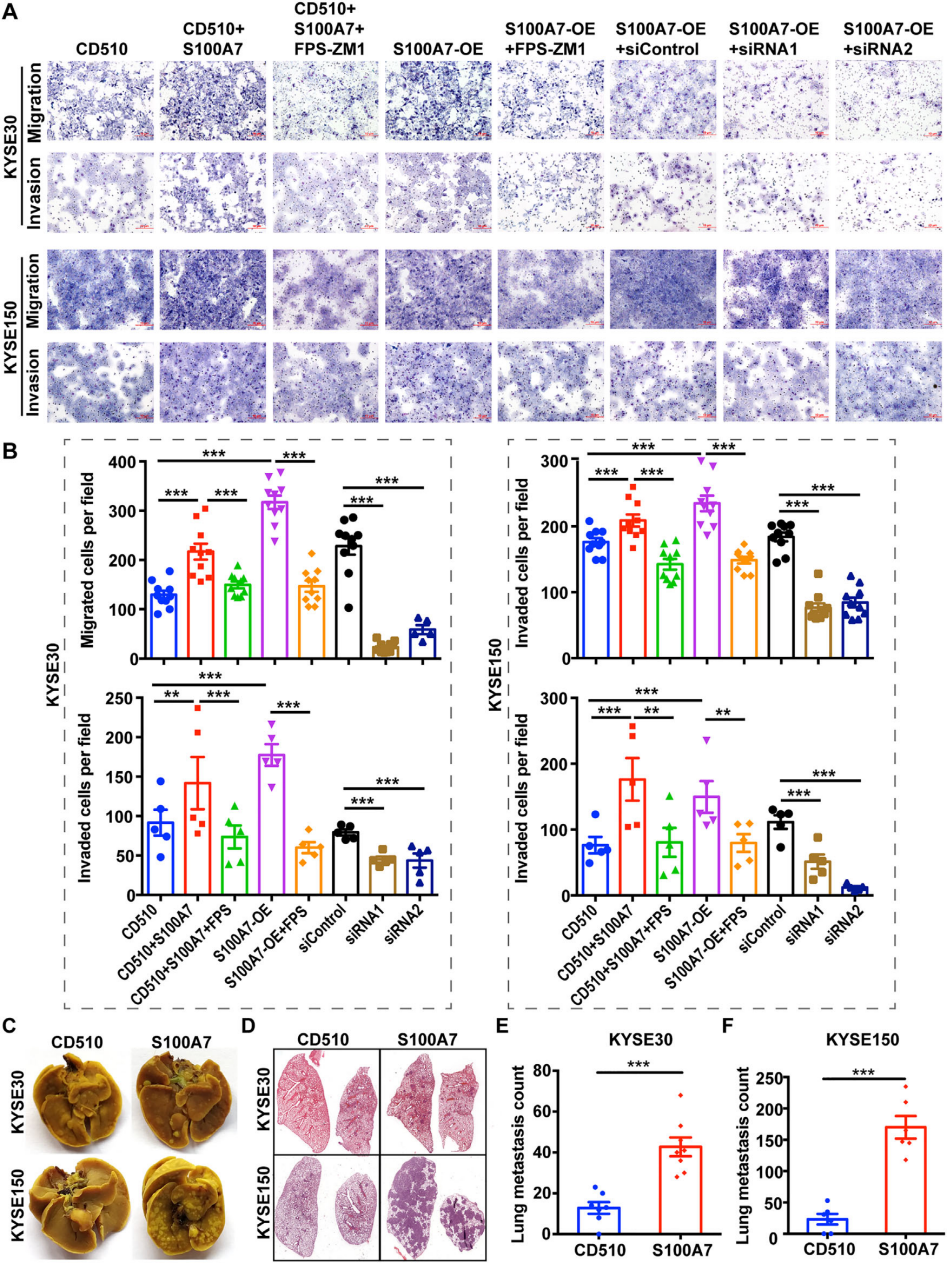
Upregulated S100A7 promotes ESCC cell metastasis in vitro and in vivo. (A, B) The migration and invasion capacities of the indicated ESCC cell clones (KYSE‐30 and KYSE‐150) treated with recombinant S100A7 protein or FPS‐ZM1 were determined using the in vitro Transwell system. Representative photographs (magnification, 100×) of migratory or invading cells on the membrane coated with or without Matrigel were presented. (C) Representative images of lung metastasis in mice after tail vein injection of indicated cells. (D) Representative images of hematoxylin and eosin staining of lung metastasis. (E, F) The number of metastatic nodules in each indicated group. **P < .01, ***P < .001

The signaling pathways regulated by S100A7 are widely involved in local invasion and distant metastasis, which are one of the 10 hallmarks of cancer and a major cause of cancer‐related death.^25^ To further investigate the effect of S100A7 on ESCC metastasis, we performed Transwell assays to characterize cancer cell migration and invasion abilities in vitro. KYSE‐30 and KYSE‐150 cells overexpressing S100A7 or treated with exogenous S100A7 protein had significantly increased invasion and migration abilities, which were at least partly abrogated by S100A7 silencing or treatment with RAGE receptor inhibitor (Figure 5A,B). Consistent with the in vitro cellular experiments, mouse lung colonization assays further confirmed the prometastasis effect of S100A7 on ESCC cells. Tail vein injection with S100A7‐overexpressing ESCC cells in mice resulted in significantly more lung metastases than injection with control ESCC cells (Figure 5C,F). Those results suggested that S100A7 plays a prometastasis role in ESCC.

### 2.6 Extracellular secreted S100A7 chemotactically recruits macrophages and promotes M2 polarization

**FIGURE 6:**
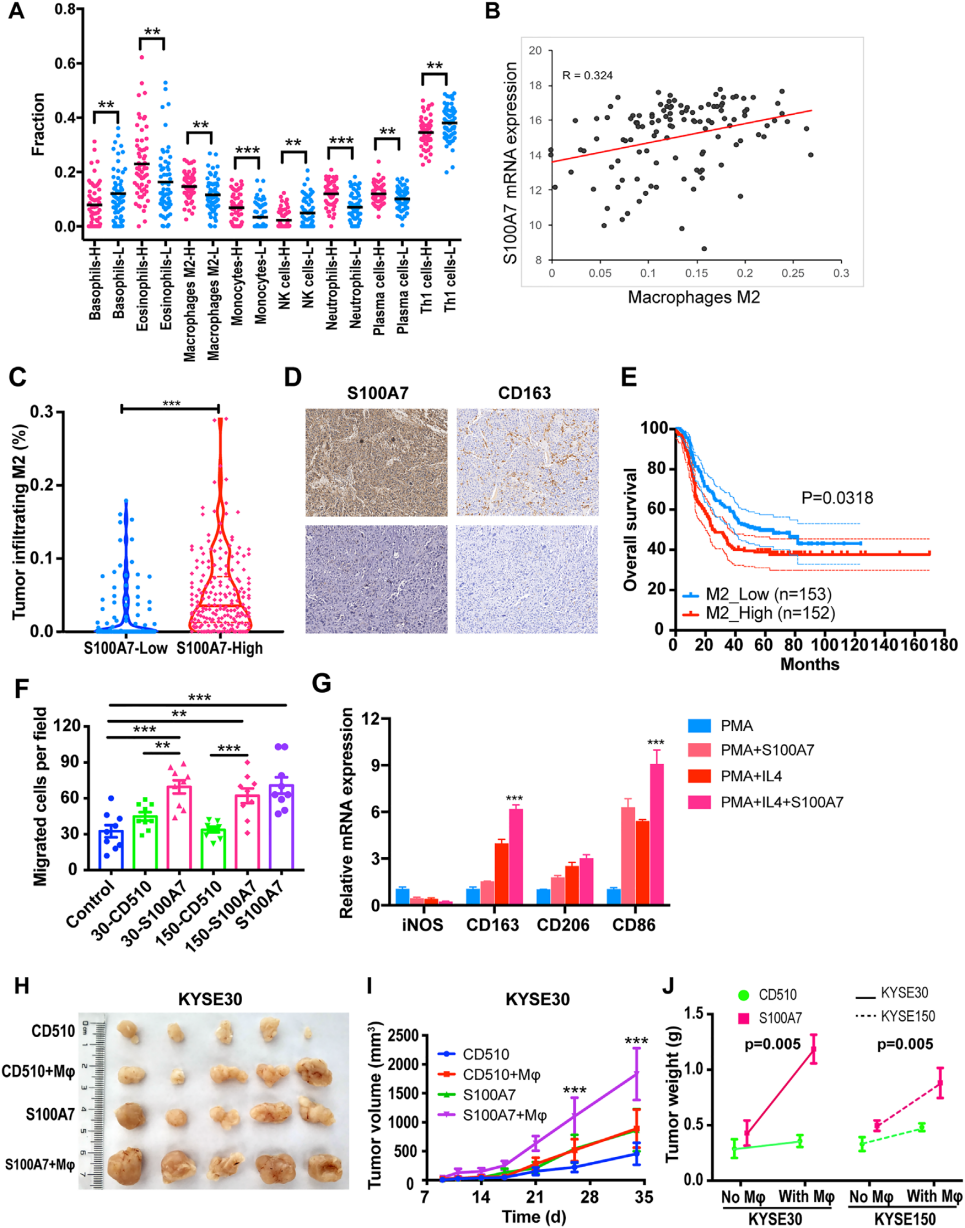
Upregulated S100A7 promotes tumor‐associated macrophage infiltration. (A) Differentially infiltrated immune cells in 119 patients with ESCC were analyzed using the xCell online tool. (B) Correlation analysis of the expression level of S100A7 mRNA and the number of infiltrating M2 macrophages. (C) Immunohistochemical analysis of M2 macrophage infiltration in ESCC tissues, as indicated by the specific molecular marker CD163. (D) Representative images of CD163‐positive cells in patients with high and low S100A7 expression. (E) Survival curve of M2 macrophage infiltration. (F) Numbers of migrated PMA‐activated macrophages under the indicated chemotactic conditions. (G) RT‐qPCR was used to measure the expression levels of M2 macrophage‐specific markers in PMA‐activated THP1 cells induced by IL4 and/or S100A7. (H) Tumor nodes of S100A7 overexpressing and vector‐control KYSE‐30 clones mixed with or without activated macrophages (Mφ). (I) Subcutaneous xenograft growth curves of the indicated groups. (J) Interactive analysis of the synergistic effect of S100A7 overexpression and macrophage infiltration on tumor cell growth **P < .01, ***P < .001

In addition to classical tumor‐related pathways, many immune‐related signaling pathways were also enriched among the genes that were differentially expressed between wild‐type ESCC cells and S100A7‐overexpressing ESCC cells (Figures 3F and S1). Those results suggested that S100A7 participates in the regulation of the immune microenvironment of ESCC. We used the xCell online tool^26^ to analyze microarray data of 119 pairs of ESCC tissues (GSE53625) and found that the expression of S100A7 was correlated with the infiltration of various immune cells. In particular, the infiltration of M2 macrophages in the tumor microenvironment was positively correlated with the expression level of S100A7 (Figures 6A,B and S3A, S3B, S3C). Immunohistochemical analysis of 306 ESCC tissue microarrays using anti‐CD163 antibodies, a molecular marker of M2 macrophages, revealed that the infiltration of M2 macrophages was increased in ESCC tissues with high S100A7 expression compared with that ESCC tissues with low S100A7 expression (Figure 6C,D). Furthermore, the patients with higher M2 macrophage infiltration had shorter overall survival than those with less M2 macrophage infiltration (Figure 6E). We performed a chemotaxis assay using the Transwell chamber system and found that treatment with exogenous S100A7 protein or S100A7 overexpression in ESCC cells had chemotactic effects on PMA‐activated THP‐1 cells (Figure 6F). Similarly, mouse S100a7a protein was capable of stimulating chemotaxis in the murine macrophage cell line RAW264.7 (Figure S3D). In addition, S100A7 upregulated the expression of M2 macrophage‐associated markers induced by IL4 (Figure 6G), suggesting that S100A7 promotes M2 macrophage polarization.

To further investigate the function of the interaction between tumor cells and macrophages mediated by secreted S100A7, we mixed S100A7‐overexpressing or control ESCC cells with PMA‐activated THP‐1 cells and injected them subcutaneously into mice. We observed that KYSE‐30 and KYSE‐150 cells formed larger tumor nodules when mixed with macrophages than when they were injected without macrophages (Figures 6H,I and S3E, S3F). Moreover, interaction analysis revealed significant synergy between S100A7 upregulation and mixing of the ESCC cells with macrophages (Figure 6J). Those results suggested that, in addition to directly affecting tumor cells, secreted S100A7 promotes the progress of tumor cells indirectly by interacting with macrophages.

### 2.7 Extracellular secreted S100A7 promotes angiogenesis

**FIGURE 7:**
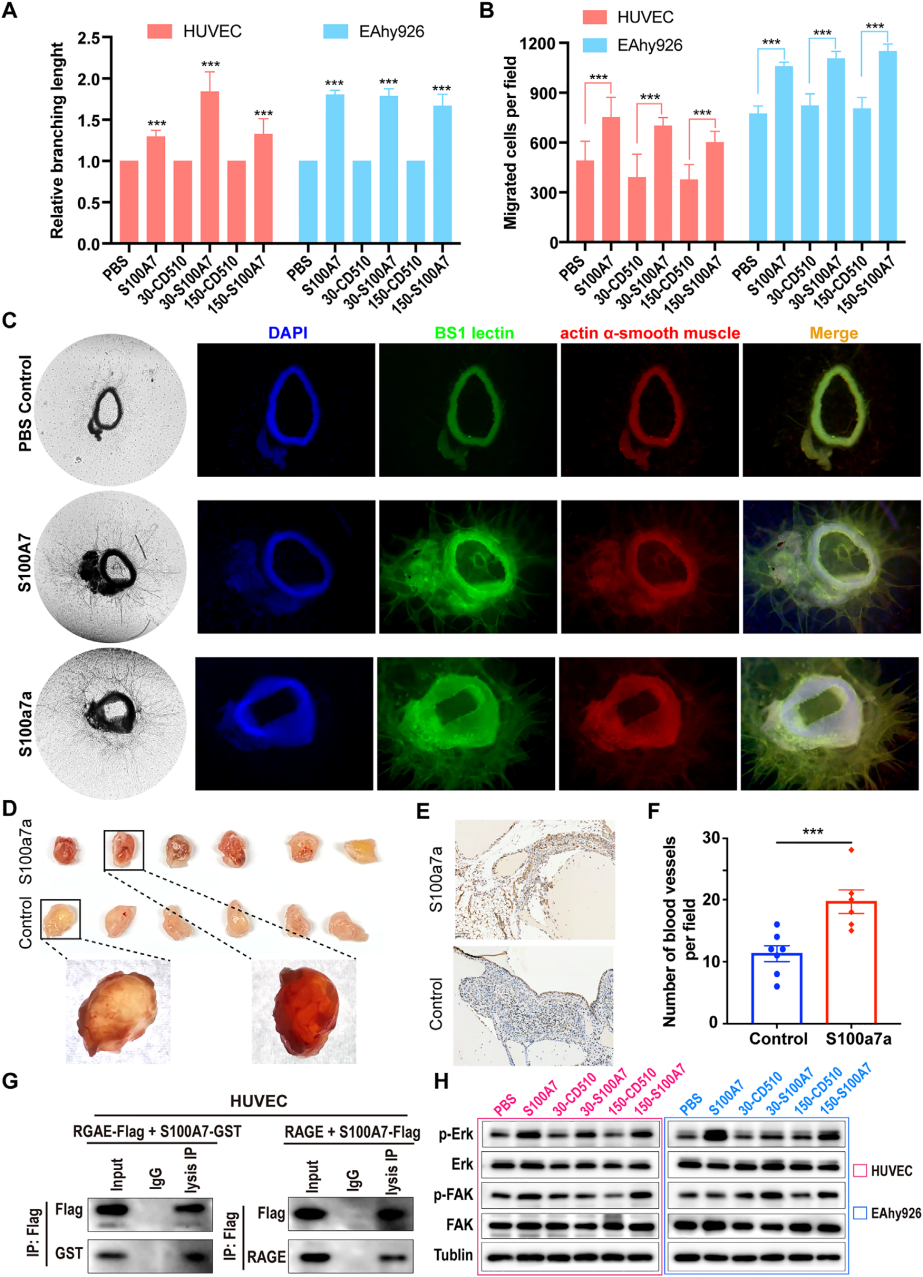
Extracellular secreted S100A7 promotes angiogenesis in vitro and in vivo. (A) The branching lengths of HUVECs and EAhy926 cells in tube formation assays under different culture conditions. (B) Effects of S100A7 protein and culture supernatant of S100A7 overexpressing ESCC cells on the migration ability of vascular endothelial cells. (C) Representative images of neo‐microvessels of mouse aortic rings treated with recombinant murine S100a7a, human S100A7, or PBS as a control. Bright field and immunofluorescence images are shown on the left and right, respectively. (D) Images of excised Matrigel nodules at the end of the in vivo Matrigel plug assay. (E) Representative images of blood vessels detected by CD31 staining of S100a7a‐treated and control groups. (F) Graphs show the vascular density of the excised Matrigel nodules estimated by CD31 staining. (G) Coimmunoprecipitation of S100A7 protein and RAGE receptor on the membrane of HUVECs. (H) The phosphorylation levels of ERK and FAK in HUVECs and EAhy926 cells, determined by western blot. ***P < .001

In addition to the inherent invasion and migration abilities of tumor cells, tumor metastasis is the result of interaction between cancer cells and the surrounding tumor microenvironment, in which angiogenesis is one of the most important factors.^27^ Moreover, increased tumor‐associated macrophages in the tumor microenvironment can promote cancer angiogenesis.^28^ Therefore, we explored the effect of exocrine S100A7 protein on tube formation by vascular endothelial cells and found that treatment with S100A7 protein or supernatant from cultures of S100A7‐overexpressing cells enhanced the tube‐forming ability of endothelial cells (Figures 7A and S4A). In addition, the migration ability of vascular endothelial cells, another important factor in angiogenesis, was facilitated by treatment with S100A7 protein or supernatant from S100A7‐overexpressing cells (Figures 7B and S4B). To further investigate the effect of S100A7 on angiogenesis, we performed aortic arch angiogenesis assays. The results showed that S100a7a, the mouse homolog of human S100A7, significantly promoted neovascularization in mouse aortic rings (Figure 7C). Treatment with human recombinant S100A7 protein also promoted microvessel formation in mouse aortic rings (Figure 7C), suggesting that the human S100A7 protein and the homologous mouse protein S100a7a are sometimes functionally interchangeable. Similar results were obtained in Matrigel plug assays in vivo; compared with injection of Matrigel alone as a control, subcutaneous injection of Matrigel containing recombinant murine S100a7a protein resulted in increased formation of new blood vessels (Figure 7D‐F). Previous studies confirmed that secreted S100A7 can bind to the RAGE receptor on the membrane of macrophages, which is also expressed on vascular endothelial cells. To determine if the proangiogenesis effect of S100A7 depends on the RAGE receptor, we incubated S100A7 protein with RAGE‐overexpressing human umbilical vein endothelial cells (HUVECs) for coimmunoprecipitation assays. As shown in Figure 7G, S100A7 could bind with RAGE receptor on the HUVEC outer membrane, which suggested that S100A7 secreted by cancer cells might promote angiogenesis by activating the RAGE pathway. We then detected the downstream signaling and found that both S100A7 protein and supernatant of S100A7‐overexpressing ESCC cells could promote the phosphorylation of ERK and FAK (Figure 7H), which are vital signals for angiogenesis. Those findings suggested that extracellular secreted S100A7 promotes angiogenesis by interacting with the tumor microenvironment.

## 3 DISCUSSION

Emerging data have implicated the importance of S100A7 in the pathogenesis of various human disorders, including cancers. Our study demonstrates that S100A7, a member of the S100 protein family, was upregulated in the tissues and blood of patients with ESCC, making S100A7 a potential diagnostic and prognostic indicator. Upregulated S100A7 promoted cell migration and proliferation through intracellular binding to JAB1 as well as through secretion and intercellular activation of RAGE receptors. In addition, extracellular S100A7 promoted infiltration of M2 macrophages in the tumor microenvironment and angiogenesis through interaction with the RAGE receptor of vascular endothelial cells.

In the past few decades, progress in the treatment of ESCC has been very limited. Because of a lack of effective diagnostic and prognostic biomarkers, most patients with ESCC are not diagnosed until the disease is in an advanced stage, leading to an overall five‐year survival rate of less than 30%.^1^ Therefore, it is urgent to learn more about the underlying molecular mechanisms of ESCC progression and develop effective diagnostic and prognostic markers. S100A7 was first identified as a highly abundant cytoplasmic and secretory protein that is induced in abnormally differentiated squamous epithelial cells of psoriatic epidermis.^9^ In recent years, a number of studies demonstrated that the dysregulation of S100A7 was a common feature in various cancer cells, especially in breast cancer,^29^ oral squamous cell carcinoma,^17^, ^30^ gastric cancer,^31^ and lung cancer.^32^, ^33^ Despite those insights, the role of S100A7 in ESCC is still poorly understood. We found that S100A7 was remarkably upregulated in ESCC and performed well as an independent prognostic factor. In addition, numerous studies have reported that S100A7 can not only function within the cell but can also be secreted and detected in serum (lung squamous cell carcinoma),^34^ saliva (oral squamous cell carcinoma),^35^ urine (melanoma),^36^ and other body fluids. Moreover, S100A7 was upregulated in the serum of patients with lung squamous cell carcinoma.^34^ On the basis of those findings, we hypothesized that S100A7 levels would be increased in the blood of patients with ESCC. ELISA to detect S100A7 in serum revealed not only that the serum S100A7 level was dramatically increased in patients with ESCC but also that S100A7 had good diagnostic efficacy to distinguish patients with ESCC from healthy individuals. Moreover, combination of S100A7 with the diagnostic biomarkers SCC and CRFRA21‐1 significantly improved the ability to effectively diagnose ESCC. Because of the heterogeneity of cancers, a single blood marker is not enough to obtain ideal diagnostic efficiency. Our results showed that a three‐marker diagnostic model for ESCC had better diagnostic performance than a two‐marker model; however, further cohort studies and prospective studies are needed to explore the potential for clinical application of S100A7 as a diagnostic biomarker.

Several studies have described a crucial role for intracellular and extracellular S100A7 in certain cancers; nevertheless, the function and mechanism of S100A7 in ESCC remain uncharted territory. Our results revealed that intracellular S100A7 plays an oncogenic role in ESCC by activating cell proliferation and promoting metastasis, which is consistent with previous findings in breast invasive carcinoma (BRCA), oral squamous cell carcinoma (OSCC), and nonsmall cell lung cancer cells (NSCLC).^18^, ^37^ Exploration of the underlying molecular mechanisms demonstrated that intracellular S100A7 binds to JAB1 to promote the activation of the cancer‐associated AKT, ERK, and NF‐kB signaling pathways, thereby promoting ESCC progression. Furthermore, we found that the enhanced proliferation and metastasis capacity of ESCC cells brought about by S100A7 overexpression or treatment with exogenous S100A7 could be moderated by pretreatment with RAGE inhibitors, which suggested the presence of an S100A7‐RAGE autocrine loop. However, rather than binding to a single specific ligand or even a group of closely related ligands, RAGE binds to several classes of molecules that lack sequence similarities. Among them, HMGB1 (High Mobility Group Box 1) is well known as another important ligand for tumor progression.^38^ Therefore, we first investigated the correlation between S100A7 and HMGB1 using the transcriptome data from TCGA database, GSE53622 and GSE23400. The analysis revealed a strong negative correlation between S100A7 and HMGB1 (TCGA: *r* = –0.423, *P* < .001, Figure S5A; GSE53622: *r* = –0.314, *P* = .014, Figure S5B; GSE23400: *r* = –0.306, *P* = .026, Figure S5C). In addition, western blot analysis showed that there was no difference of HMGB1 expression between S100A7‐overexpressing ESCC cells and control cells (Figure S5D). Therefore, although the HMGB1/RAGE axis plays an important role in the progression, metastasis, autophagy, and chemoresistance of many malignancies. However, our results revealed that S100A7/RAGE axis may play the same or stronger role in the tumor progression and metastasis of ESCC when the influence HMGB1 was absent. Several previous studies also found that many S100A7 functions are RAGE dependent and activate downstream signaling molecules such as AP‐1, an NF‐κB, and STAT3(14) to promote cancer pathogenesis. Our results along with the previous results suggest that S100A7 promotes oncogenic signal activation in ESCC cells by direct binding to JAB1 and paracrine interaction.

In recent decades, researchers gradually began to pay more attention to the tumor microenvironment and came to understand that cancer progression is the result of interactions between cancer cells, surrounding stromal cells and immune cells, rather than simply the accumulation of mutations in tumor cells.^39^, ^40^ Cancer cells and stromal cells in the tumor microenvironment communicate with each other through effector molecules such as cytokines and chemokines in a paracrine or autocrine manner. S100A7, as an exocrine protein, was reported to increase the recruitment of tumor‐associated macrophages,^41^ whose infiltration is associated with poor prognosis and chemotherapy resistance in most cancers, including ESCC.^28^, ^42^ Indeed, tumor‐associated macrophages can have a dual supportive and inhibitory influence on cancer, depending on their polarization to type M1 or type M2. M2 macrophages are generally thought to promote tumor progression by increasing tumor cell migration, invasion, and intravasation metastasis; stimulating angiogenesis; and suppressing antitumor immunity. We demonstrated that the expression of S100A7 was correlated with M2 macrophage infiltration and poor prognosis in ESCC. In vitro experiments showed that S100A7 could chemoattract macrophages and promote the expression of M2 polarization markers. The interaction between overexpressed S100A7 and macrophages synergistically promoted tumor growth in vivo. S100A7 has also been reported to promote angiogenesis by regulating cytokines. In our study, we confirmed the promoting effect of S100A7 on multiple aspects of angiogenesis in vitro and in vivo. Mechanistically, we found that the combination of exocrine S100A7 and RAGE on endothelial cells activated ERK and FAK signals, thereby promoting angiogenesis. Overall, our results demonstrate that S100A7 functions in a highly complex manner involving both intracellular and extracellular interactions, making it difficult to clarify its full molecular mechanisms in detail. We speculate that S100A7 acts as a central mediator of tumor invasion, matrix remodeling, and angiogenesis, leading to a more aggressive milieu, which in turn supports tumor progression and facilitates metastatic spread. In summary, we demonstrated an important role of S100A7 in ESCC and its implications for the development of new diagnostic methods and treatment strategies for ESCC.

## 4 METHODS AND MATERIALS

### 4.1 Human tissues and serum samples

All human tissues and serum samples were collected from the Cancer Hospital, Chinese Academy of Medical Sciences. ESCC tissues and adjacent nontumor esophageal tissues for tissue arrays were obtained from 341 patients that underwent radical resection from December 2005 to December 2008. Additional serum samples from 274 patients with ESCC and 151 healthy controls were obtained from Laboratory Department during May 2018 to December 2019. All cases of ESCC were pathologically confirmed. None of the patients with ESCC had received antitumor therapy or had a history of other malignancies within the three years prior to their ESCC diagnosis. The clinicopathological characteristics of the patients were collected through telephone interviews and routine laboratory studies conducted prior to surgery. Tumor and adjacent normal tissues obtained during surgery were snap frozen in liquid nitrogen within 30 minutes of resection and stored at –80°C until the tissue arrays were manufactured. Serum samples were aliquoted into 200 μL portions, which were stored in separate tubes at –80°C until use. Exclusion criteria for the study were incomplete medical records, hemolysis, and exposure of a given sample to repeated freeze‐thaw cycles. The Ethics Review Committee of Research Involving Human Subjects of the Cancer Hospital, Chinese Academy of Medical Sciences granted our ethics approval (20/287‐2483).

### 4.2 Aortic arch angiogenesis assay

Aortic arch angiogenesis assays were performed according to a previous report.^43^ Briefly, 5‐week‐old C57/BL6 mice were sacrificed and dissected to remove the thoracic aorta. The dissected aortae were immersed in Opti‐MEM and cut into rings ∼0.1‐0.5 mm in width and transferred to a 10 cm dish containing Opti‐MEM. The rings were serum‐starved overnight in a cell incubator and then embedded separately in a Slide‐chamber well (BIOLOGIX) with Collagen type I (Millipore, #08‐115). Add 150 μL of Opti‐MEM medium supplemented with penicillin‐streptomycin, 2.5% (vol/vol) FBS and VEGF (30 ng/mL) to the embedded rings. Recombinant human S100A7 protein or murine S100a7a protein was then added to the medium. Next, the arterial rings were divided into three groups for treatment with S100A7 (100 ng/mL, R&D), S100a7a (100 ng/mL, Origene), and PBS, respectively. After the treatments were added to the cultures, the growth medium was changed first on day 3 after the start of the treatments and then every other day.

Microvessel growth during the experiment was quantified by live phase‐contrast microscopy. On day 7, the culture medium was removed, and the whole plates were washed with wash buffer A (PBS, CaCl_2_, MgCl_2_). The cells in each well were fixed with 4% formalin for 30 minutes at room temperature, permeabilized by with buffer (PBS, CaCl_2_, MgCl_2_, 0.25%(vol/vol) Triton X‐100) for 15‐minute incubations at room temperature (twice), and then blocked with 2% fetal bovine serum (FBS) for 30 minutes at 37°C. The following primary antibodies were prepared in PBLEC solution: BS1 lectin‐FITC (Sigma, #L9381) to label endothelial cells and antiactin α‐smooth muscle Cy3 (Sigma, #C6198)) for supporting cells. The permeabilized and blocked cells were incubated overnight with antibody solution at 4°C, washed by buffer C (PBS, 0.1% (vol/vol) Triton X‐100), and then sealed with antifade mounting medium with DAPI (Thermo #P36966). The stained neo‐microvessels were then analyzed with a fluorescence microscope.

### 4.3 In vivo animal experiments

All mice were purchased from Huafukang Bioscience (Beijing, China) and housed at the SPF Animal Experiment Center, Cancer Hospital, Chinese Academy of Medical Sciences. All animal experimental procedures were approved by the Animal Care and Use Committee.

#### 4.3.1 Subcutaneous xenograft assay

KYSE‐30 and KYSE‐150 cells with S100A7‐overexpressing and mock vehicle‐control were injected (1 × 10^6^ cells) into the right flank subcutaneously of 4‐week‐old nude mice. Tumor length (a) and minor diameter (b) were measured twice or three times a week. Tumor volume was calculated using the formula *V* = *ab* ^2^/2. When the tumor volume reached the ethical limit (1000 mm^3^), the mice were euthanized, and the tumors were harvested, weighed, and photographed. The tumor nodules were then fixed with formalin, with hematoxylin and eosin (H&E) or immunohistochemical stains.

#### 4.3.2 Lung colonization assay

S100A7‐overexpressing and mock vehicle‐control KYSE‐30 and KYSE‐150 cells were injected (1 × 10^6^ cells) into the tail vein of 4‐week‐old female NOD/SCID‐beige mice. The mice were sacrificed using CO_2_ anesthesia seven weeks later. The integral lungs were excised for subsequent picric acid staining, formalin fixation, paraffin embedding, and H&E staining. The numbers of tumor nodules on the lungs were then counted.

*Macrophage mixed xenograft model*. ESCC cells were mixed with PMA‐activated macrophages and injected subcutaneously into the axilla of the right upper limb of 4‐week‐old nude mice. The volume of the tumor nodules was measured periodically as described in the subcutaneous xenograft assay. The mice were sacrificed after 5 weeks.

### 4.4 Statistics

GraphPad Prism 8 (GraphPad Software, Inc.) and SPSS 23 (IBM, USA) were used for data analysis. Student's *t*‐test was used for comparisons between two groups. Mann‐Whitney *U* test was used to compare nonnormally distributed data. Pearson's chi‐square test was used for clinicopathological correlation analysis. Kaplan‐Meier analysis and Cox regression were performed for univariate and multivariate prognosis analysis, respectively. Correlation coefficients were obtained by Pearson correlation analysis. Logistic regression analysis was carried out to integrate multiple diagnostic markers. Two‐sided test was used for all statistical analysis, and *P* ‐values < .05 were considered statistically significant. The graphical abstract was created with BioRender.com.

The details of other experimental materials and methods can be found in the Supplementary Materials

## AUTHOR CONTRIBUTIONS

LZL, ZSF, LCM, SN, and HJ designed and supervised the study; LZL, ZSF, and LCM performed the most of experiments; WXF, ZGC, WF, WSH, and LYJ were responsible for the clinical sample collection and analysis; MSS and HJB took charge of bioinformatics and statistical analyses; HJB and LYY provided technique supports; LZL, ZSF, and LCM undertook data analysis and manuscript writing.

## ETHICS APPROVAL AND CONSENT TO PARTICIPATE

Written informed consent for individual patients was gained from all participants and can be provided upon request. All the collection of samples and animal operation in this study were approved by the Ethics Committee of National Cancer Center/National Clinical Research for Cancer/Cancer Hospital Chinese Academy of Medical Sciences.

## CONSENT FOR PUBLICATION

Not applicable.

## COMPETING INTERESTS

The authors declare that they have no competing interests

## Supporting information


**FIGURE S1**. Functionally grouped network analysis of KEGG pathways and gene ontology functional terms enriched among genes that are differentially expressed when S100A7 is overexpressed in cells
**FIGURE S2**. S100A7 promotes cell proliferation and inhibits apoptosis. (**A**) The left side shows proliferation curves of S100A7‐overexpressing and control KYSE‐150 cells treated with or without recombinant human S100A7 protein or the RAGE‐specific inhibitor FPS‐ZM1. The right side shows the proliferation curves after S100A7 silencing in KYSE‐150‐overexpressing cells and control cells. (**B**) Flow cytometric analysis revealed that S100A7 had no effect on the cell cycle. (**C**) Representative results of flow cytometric analysis of apoptosis. (**D**) Cisplatin‐induced apoptotic cell ratio of S100A7‐overexpressing cells and vector‐containing control cells
**FIGURE S3**. The interaction of S100A7 and macrophages in the tumor microenvironment promotes cancer progression. The proportion of tumor infiltrating immune cells is shown in patients with (A) high S100A7 expression and (B) low S100A7 expression. (**C**) Heatmap showing correlation coefficients of differentially infiltrated immune cells between tumors with high S100A7 expression and tumors with low S100A7 expression. (**D**) The Transwell system was used to evaluate the chemotactic effect of S100a7a, the mouse homolog of S100A7, on the mouse macrophage cell line RAW264.7. The left side shows representative images, and the right side shows the number of migrating cells. (**E**) Representative images of migrated PMA‐activated macrophages under the indicated chemotactic conditions. (**F**) Tumor nodes of S100A7‐overexpressing and vector‐containing control KYSE‐150 clones mixed with or without activated macrophages (Mφ). (**G**) Subcutaneous xenograft growth curves of the indicated treatment groups
**FIGURE S4**. S100A7 promotes tube formation and migration of endothelial cells. (**A**) Representative pictures of tubules formed by HUVECs and EAhy926 vascular endothelial cells treated with or without S100A7 protein or the indicated culture supernatants. (**B**) Representative pictures of HUVECs and EAhy926 cells passing through chambers treated with or without S100A7 protein or the indicated culture supernatants
**FIGURE S5**. The correlation between S100A7 and HMGB1 in ESCC. The correlation between S100A7 and HMGB1 by the transcriptome data from TCGA database (A), GSE53622 (B) and GSE23400 (C). (**D**) Western blot analysis of the correlation between S100A7 and HMGB1 from vector‐control and S100A7‐overexpressing ESCC cells
**TABLE S1**. Correlation analysis of the expression of free S100A7 in serum and clinicopathological characteristics
**TABLE S2**. Primers used in this study
