# Erratum for the “S100A7 as a potential diagnostic and prognostic biomarker of esophageal squamous cell carcinoma promotes M2 macrophage infiltration and angiogenesis” by Zhiliang Lu et al.

**DOI:** 10.1002/ctm2.70222

**Published:** 2025-02-03

Lu Z, Zheng S, Liu C, et al. S100A7 as a potential diagnostic andprognostic biomarker of esophageal squamous cellcarcinoma promotes M2 macrophage infiltrationand angiogenesis. *Clin Transl Med*. 2021;11:e459. doi: 10.1002/ctm2.459

The reason for the correction:

We proofread the entire article and found that the Western Blot band of the p65 protein in the S100A7 siRNA silencing group in Figure 3H on page 6 accidentally used the same band as the p65 protein in the S100A7 siRNA silencing group in Figure 3G during the editing process.

**FIGURE 3:**
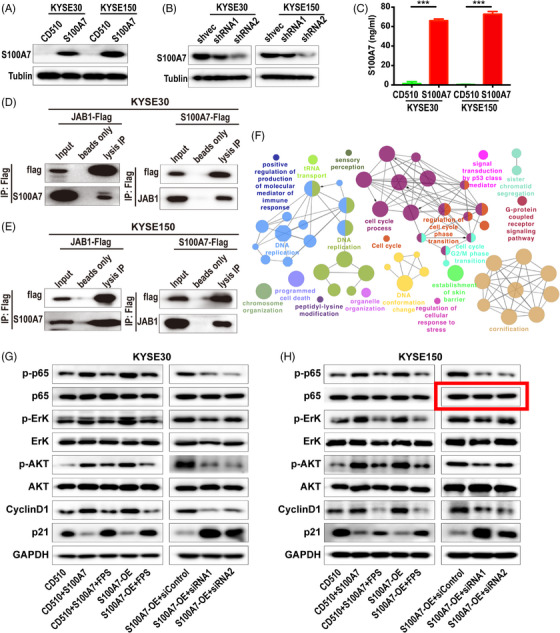
Original version.

The western Blot band of p65 protein in the S100A7 siRNA silencing group in Figure 3H that needs errata is marked with a red block below.

**FIGURE 3:**
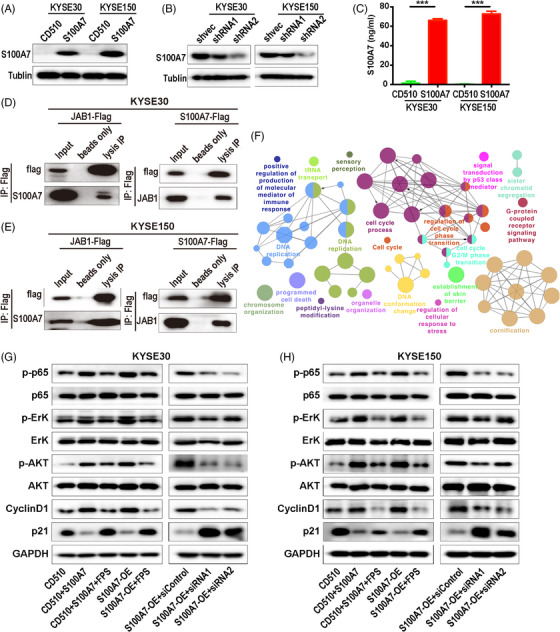
New version.

