# Supplementary material for: S100A7 as a potential diagnostic and prognostic biomarker of esophageal squamous cell carcinoma promotes M2 macrophage infiltration and angiogenesis
Source: Clin Transl Med. 2021 Jul 8;11(7):e459. doi: 10.1002/ctm2.459 (PMC8265170; doi:10.1002/ctm2.459)
Supplement: Supplementary file 1 — FIGURE S1. Functionally grouped network analysis of KEGG pathways and gene ontology functional terms enriched among genes that are differentially expressed when S100A7 is overexpressed in cells FIGURE S2. S100A7 promotes cell proliferation and inhibits apoptosis. (A) The left side shows proliferation curves of S100A7‐overexpressing and control KYSE‐150 cells treated with or without recombinant human S100A7 protein or the RAGE‐specific inhibitor FPS‐ZM1. The right side shows the proliferation curves after S100A7 silencing in KYSE‐150‐overexpressing cells and control cells. (B) Flow cytometric analysis revealed that S100A7 had no effect on the cell cycle. (C) Representative results of flow cytometric analysis of apoptosis. (D) Cisplatin‐induced apoptotic cell ratio of S100A7‐overexpressing cells and vector‐containing control cells FIGURE S3. The interaction of S100A7 and macrophages in the tumor microenvironment promotes cancer progression. The proportion of tumor infiltrating immune cells is shown in patients with (A) high S100A7 expression and (B) low S100A7 expression. (C) Heatmap showing correlation coefficients of differentially infiltrated immune cells between tumors with high S100A7 expression and tumors with low S100A7 expression. (D) The Transwell system was used to evaluate the chemotactic effect of S100a7a, the mouse homolog of S100A7, on the mouse macrophage cell line RAW264.7. The left side shows representative images, and the right side shows the number of migrating cells. (E) Representative images of migrated PMA‐activated macrophages under the indicated chemotactic conditions. (F) Tumor nodes of S100A7‐overexpressing and vector‐containing control KYSE‐150 clones mixed with or without activated macrophages (Mφ). (G) Subcutaneous xenograft growth curves of the indicated treatment groups FIGURE S4. S100A7 promotes tube formation and migration of endothelial cells. (A) Representative pictures of tubules formed by HUVECs and EAhy926 vascular endothelial cel [file CTM2-11-e459-s001.pdf]

Supplementary Materials

S100A7 as a potential diagnostic and prognostic biomarker of esophageal squamous cell carcinoma promotes M2 macrophage infiltration and angiogenesis

Zhiliang Lu[1#], Sufei Zheng[1#], Chengming Liu[1#], Xinfeng Wang[1], Guochao Zhang[1], Feng Wang[1], Sihui Wang[1], Jianbing Huang[1], Shuangshuang Mao[1], Yuanyuan Lei[1], ZhanYu Wang , Nan Sun[1*], Jie He[1*]

[1] Department of Thoracic Surgery, National Cancer Center/National Clinical Research for Cancer/Cancer Hospital, Chinese Academy of Medical Sciences and Peking Union Medical College, Beijing, 100021, China

# These authors contributed equally

[*] Corresponding authors: Jie He (prof.jiehe@gmail.com); Nan Sun (sunnan@vip.126.com)

Supplementary methods and materials

Cell lines

KYSE30 and KYSE150 cell lines were established from the human esophageal squamous cell carcinomas. KYSE30, KYSE150 and THP-1 cell lines were maintained in Roswell Park Memorial Institute 1640 (HyClone) media with 10% fetal bovine serum (Cornning) and antibiotics (100 U/ml penicillin and 100 mg/ml streptomycin) (Invitrogen). 293T, HUVEC, EAhy926 and RAW264.7 were maintained in DMEM with 10% FBS and antibiotics (100 U/ml penicillin and 100 mg/ml streptomycin). All cell lines were identified via short tandem repeat (STR) and periodic mycoplasma elimination was performed.

Plasmids and reagents

The cytokines and reagents used in this study are as follows: Human recombinant IL4 protein (R&D Systems, #204-IL, 20ng/ml), Recombinant Human M-CSF Protein (R&D Systems, #216-MC, 20ng/ml), Recombinant Human S100A7 Protein (R&D Systems, #9085-SA, 100ng/ml), Recombinant Human IL-4 Protein (R&D Systems, # 204-IL-050). GST-labeled S100A7 protein (CircuLex™, #CY-R2257), Recombinant mouse S100a7a protein (Origene, #AR50435PU-S), Cisplatin (Sigma, #P4394), PMA (Sigma, #P1585, 100g/ml). All reagents were dissolved and stored strictly according to the instructions. Human S100A7 and JAB1 cloned into pCMV6-Entry were bought from Origene (#RC204490). RAGE cDNA shuttle plasmids were purchased from Vigene Biosciences (Shandong, China). Full length of S100A7 was constructed into pCD513B-1 for stable overexpression. The specific siRNAs of S100A7 and non-targeting control siRNA were purchased from Thermo Fisher (s12420, s12421 and 13778030).

Lentiviral S100A7 stable overexpression

Full-length S100A7 cDNA was ligated into the pCDH-CMV-MCS-EF1-Puro (CD510B) vector. To produce lentivirus containing the S100A7 gene, HEK-293T cells were co-transfected with the resulting vector described above, pLP1, pLP2 and pLP/VSVG (Invitrogen) using Lipofectamine 3000 (Life Technologies) strictly according to the manufacturer's guidelines. Infectious lentiviruses were harvested at 48 h after transfection and filtered through Amicon Ultra-4 Centrifugal Filter Devices (Millipore). KYSE30 and KYSE150 cells were infected with

concentrated virus in the presence of 5μg/ml polybrene (Sigma-Aldrich) and selected with puromycin (1.5 ng/ml). The expression levels of S100A7 in the infected cells were confirmed by qRT-PCR and western blot 96 hrs after puromycin selection.

Transient transfection

SiRNAs targeted S100A7 and negative control siRNAs (Thermo Fisher) were introduced into cells with RNAiMAX (Life Technologies). For transfection in 6-well plates, 9μl transfection reagent per well was diluted in 150μl Opti-MEM(Sigma) and mixed with 25 pmol siRNA in 150μl Opti-MEM. Incubated for 5 minutes then add to the cell culture dish. The expression level of S100A7 was confirmed 72 hrs later via western blot. Transient transfection of plasmids was performed with Lipofectamine 3000 kit (Invitrogen) according to the manufacturer's instructions. The transient overexpression plasmids (5 μg/6-well plate) of S100A7, JAB1 and RAGE as well as their respective negative controls were introduced into cells. The cells were harvested at 72 hr after transfection.

RNA extraction, RT-qPCR, western blot, cell proliferation and transwell assays

RNA extraction, RT-qPCR, western blot, cell proliferation and transwell assays were performed as previous study[19]. Total RNA was obtained with TRIzol RNA-Extraction reagent according to the instructions. First-Strand cDNA Synthesis kit (Life Technologies, #K1612) was used for reverse transcription. RT-qPCR was performed with SYBR™ Select Master Mix (Life Technologies, #4472908) on ABI 7900HT Real-Time PCR thermocycler (Life Technologies). The PCR primers used in this study are shown in the table S2. Antibodies for western blot used in this study are listed as follows: anti-S100A7 (Abcam ab13680, 1:1000), anti-HMGB1 (Abcam ab79823, 1:1000), anti-JAB1 (CST #6895, 1:1000), anti-RAGE (R&D AF1145) anti-Flag (Sigma, #F1804, 0.5ug/ml WB), anti-Flag rabbit mAb (Abclonal, #AE063), anti-GST (Abclonal, #AE001, 1:5000), anti-p-p65 (CST #3033, 1:1000), anti-p65 (CST #8242, 1:1000) anti-p-Erk (CST #4370, 1:1000), anti-Erk (CST #4695, 1:1000), anti-AKT (CST #4685, 1:1000), anti-p-AKT (CST #4060, 1:1000), anti-GAPDH (Abcam ab8245, 1:5000), anti-beta Tublin (Abcam ab179513, 1:5000) anti-p21 (CST #2947, 1:1000), CD163 (CST #93498, 1:1000). Cell proliferation was quantified using the Cell Counting Kit-8(DOJINDO, CK04). The Transwell assay was performed using the Transwell chamber coated with or without Matrigel (BD Biosciences, San Jose, CA, USA).

Cell cycle and apoptosis

The cell cycle analysis was performed with Cell Cycle Detection Kit (KeyGEN BioTECH, #KGA511) according to the manufacturer's introductions. To evaluate cell apoptosis, flow cytometry was performed using the Annexin V-FITC Apoptosis Detection Kit (KeyGEN BioTECH, #KGA107). Treatment with 5μM cisplatin for 24 hours was used to induce apoptosis.

Immunohistochemistry (IHC)

IHC was performed as described previously. The expression level of S100A7 protein in 331 ESCC tissues and adjacent esophagus tissues were analyzed using IHC via anti-S100A7 antibodies (abcam, #ab13680, 1:100). Tumor infiltrated M2 macrophages were evaluated via analyzing the expression level of CD163 (CST #93498, 1:500). The expression level of Ki67 (CST #9449), PCNA (CST #2586) and p21 (CST #2947) in mouse xenografts were detected via IHC analysis to evaluate the tumor growth rate indirectly. IHC results were interpreted by pathologists, which was carried out blindly to the clinical data. The Immunohistochemical Score = Staining Area (less than 30% is 1 point. 30% to 60% is 2 points, more than 60% is 3 points) ×Staining Intensity (no expression is 0, weak positive is 1 point, medium positive is 2 points, strong positive is 3 points). When the Immunohistochemical Score was 0, 1-3, 4-5, 6-9, they were divided into -, +, ++, +++ groups.

Enzyme linked immunosorbent assay (ELISA)

The concentration of free S100A7 protein in serum of ESCC patients and control people was measured by CircuLex S100A7/Psoriasin ELISA Kit (#CY-8073) according with the instructions. For each well 100μl serum without dilution was used for the ELISA analysis of S100A7. Two biological duplicates were designed for each sample. The optical densities (OD) were measured immediately after adding stop solution at 450 nm using a spectrophotometer (SpectraMax® 190, Molecular Devices, Sunnyvale, CA, USA).

THP-1 macrophage differentiation and polarization

THP-1 cells were plated on FBS coated surfaces at $1x10^5$ cells/cm^2 in RPMI-1640/10% FBS plus 100ng/mL PMA (Sigma) for 72hrs to differentiate into M0-macrophages. M2 polarization by 20 ng/mL of recombinant human IL- 4 (R&D) for another 4 days.

Cell chemotaxis assay

The chemotaxis experiment was implemented by Transwell chamber. PMA activated THP-1

M0-macrophages were seeded on the upper chamber with FBS free media. S100A7 overexpressing cells, control cells, 100ng/ml S100A7 protein and control medium were added to the lower chamber. The culture media added in the lower chamber was 1640 with zinc ions and 1% FBS. Twenty-four hours later, cells on the lower side of the chamber were fixed, stained and counted in five different areas at 100-fold magnification.

Murine bone marrow-derived macrophage (BMDM) isolation and polarization

Bone marrow (BM) was flushed from femurs and tibia of wild type (WT) C57BL/6 mice. Filtered the cells through a 200 mesh nylon mesh into a 15 ml centrifuge tube and centrifuge at 2000 rpm for 5 minutes; discard the supernatant, resuspend in erythrocyte lysate (KeyGEN BioTECH, #KGP11100) and lyse at 4℃ for 10 minutes; prepare cell suspension with a final concentration of 2×10^6/ml. BM cells were cultured in DMEM containing 10% FBS, 10 ng/ml murine recombinant M-CSF. Change culture medium every 2-3days to generate mature M0 macrophages. On day 7, M1-phenotype was induced by 100 ng/mL recombinant murine LPS and 20 ng/mL IFNγ, whereas M2-polarization by 20 ng/mL recombinant murine IL-4 for another 48 hours.

Tube formation assay

The μ-Slide Angiogenesis (ibidi #81506) was used to investigate angiogenesis in tube formation assays. Fill the inner well with 10μl growth factor reduced Matrigel (BD Biosciences) and given 30min to solidify. Apply 50 μl of HUVEC or EAhy926 cell suspension into the upper well. Cover the μ-Slide Angiogenesis with the supplied lid. Incubate at 37°C and 5% CO_2 as usual. Cells were treated with 100 ng/ml S100A7 protein (R&D), indicated cell culture supernatant or PBS control. Images were captured under phase contrast microscopy. Five microscopic fields were randomly selected for each well, and the number of branch points of the tubes per field was counted. Images were automatically analyzed in ImageJ with the angiogenesis analyzer plug-in.

In vivo Matrigel plug assay.

Growth Factor Reduced Matrigel Matrix phenol Red-free (BD Biosciences) with 100ng/ml recombinant murine S100a7a protein and Matrigel blank control were injected subcutaneously into 6-week-old C57/BL6 mice. On day 7, the mice were sacrificed and Matrigels were completely excised to assess the angiogenic response. The excised Matrigel nodules were

soaked with picric acid and embedded in paraffin for hematoxylin and eosin (H&E) staining and immunohistochemistry. The levels of angiogenesis determined via IHC analysis of endothelial marker CD31 (anti-CD31, CST #3528), and count the number of new blood vessels under the microscope.

Co-immunoprecipitation (Co-IP) Assay

For the JAB1-S100A7 interaction, JAB1-Flag and S100A7-Flag overexpression plasmids were transfected into KYSE30 cells. After 48 hours, IP Lysis Buffer (Thermo Scientific, #87787) was used to harvest cell lysates. For free S100A7 protein binding to cell membrane RAGE receptor, the recombinant human S100A7-GST protein and S100A7-Flag protein were added to RAGE-Flag or RAGE overexpressed HUVEC respectively, and harvested 4 hours later. Prepared cell lysates were incubated with washed Anti-Flag M2 Magnetic Beads (Millipore #M8823). For the negative control, add 1 ml of lysis buffer. Agitated all samples and controls gently overnight at 4°C. Separated the beads-binding protein with a magnetic separator and the samples and control were eluted with 2x SDS-PAGE sample buffer. Heated to denature the eluted samples and analyzed by western blot.

Supplementary Figures

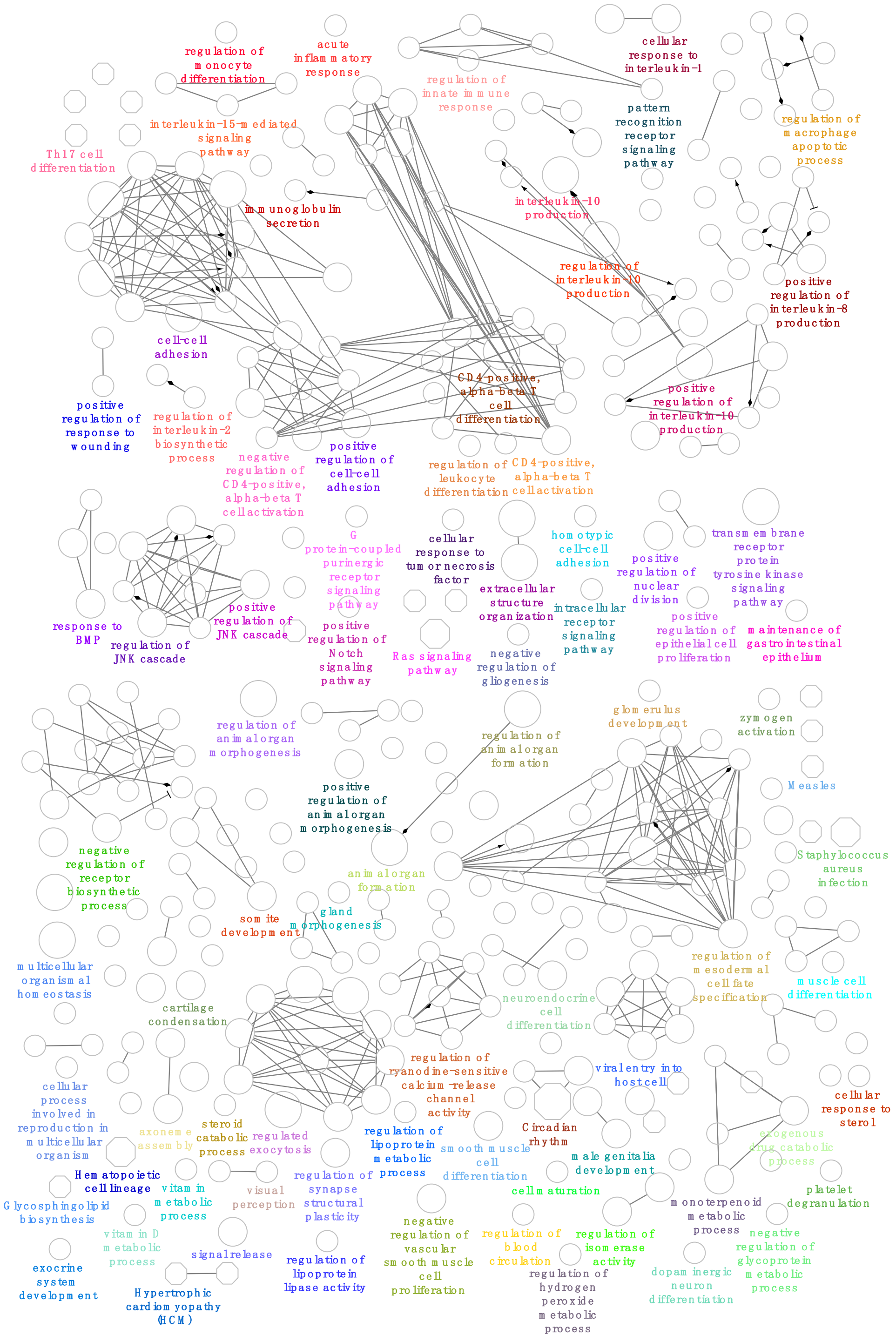

Figure S1. Functionally grouped network analysis of KEGG pathways and gene ontology functional terms enriched among genes that are differentially expressed when S100A7 is overexpressed in cells. The KEGG pathway and GO biological functional terms were enriched

by the Cytoscape Cluego plugin. Diamond, KEGG pathway. Ellipse, GO term. The node size represents the significance of the term enrichment. Different colors represent different functional groups.

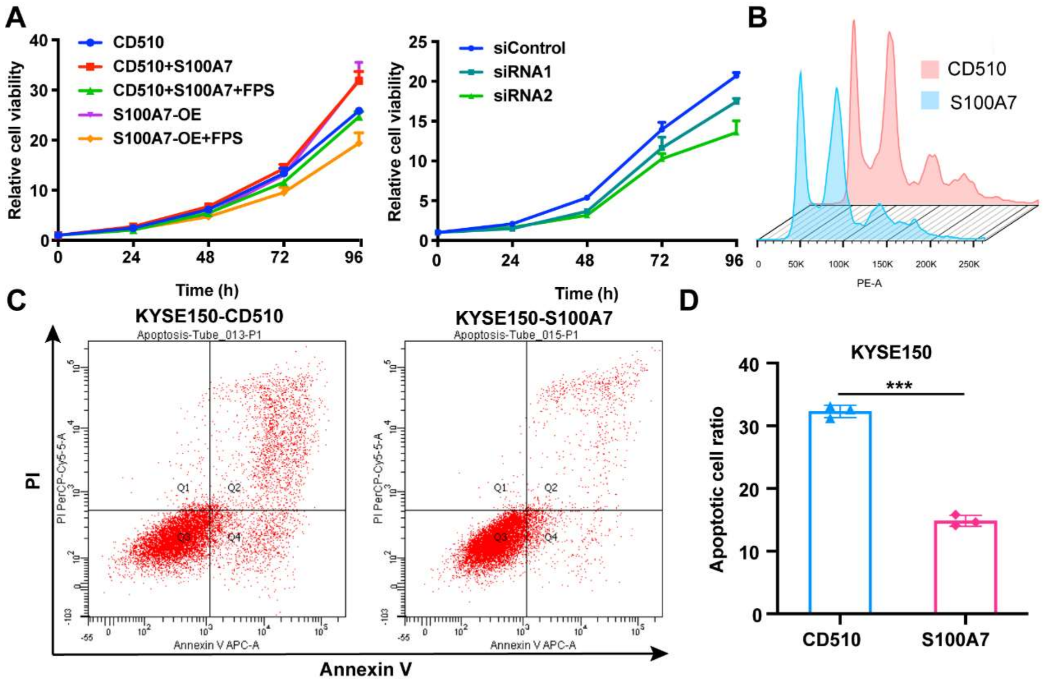

Figure S2 S100A7 promotes cell proliferation and inhibits apoptosis. (**A**) The left side shows proliferation curves of S100A7-overexpressing and control KYSE150 cells treated with or without recombinant human S100A7 protein or the RAGE-specific inhibitor FPS-ZM1. The right side shows the proliferation curves after S100A7 silencing in KYSE150-overexpressing cells and control cells. (**B**) Flow cytometric analysis revealed that S100A7 had no effect on the cell cycle. (**C**) Representative results of flow cytometric analysis of apoptosis. (**D**) Cisplatin-induced apoptotic cell ratio of S100A7-overexpressing cells and vector-containing control cells. *** $p<0.001$.

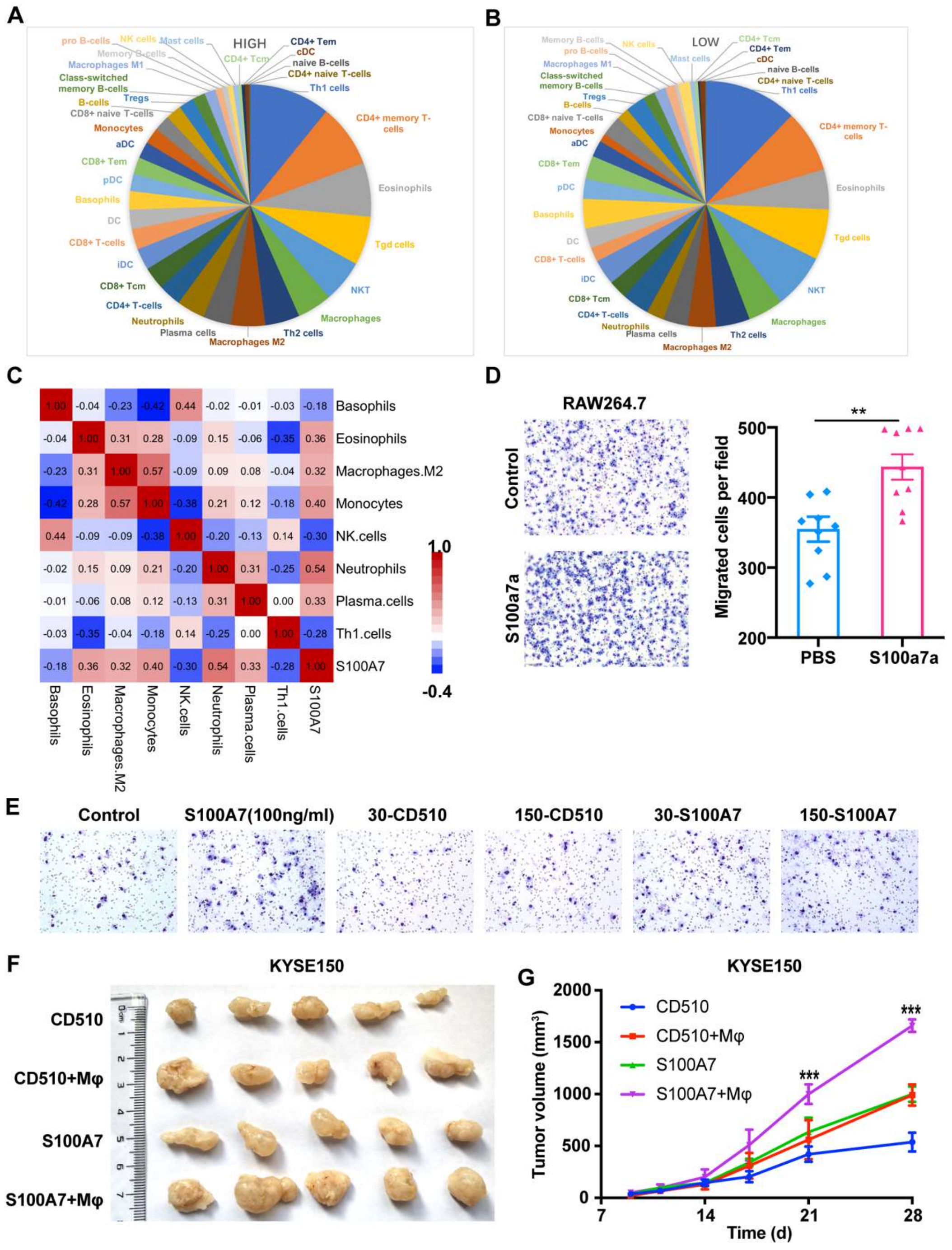

Figure S3. The interaction of S100A7 and macrophages in the tumor microenvironment promotes cancer progression. (**A** and **B**) The proportion of tumor infiltrating immune cells is shown in patients with (A) high S100A7 expression and (B) low S100A7 expression. (**C**) Heatmap showing correlation coefficients of differentially infiltrated immune cells between tumors with high S100A7 expression and tumors with low S100A7 expression. (**D**) The Transwell system was used to evaluate the chemotactic effect of S100a7a, the mouse homolog

of S100A7, on the mouse macrophage cell line RAW264.7. The left side shows representative images, and the right side shows the number of migrating cells. (**E**) Representative images of migrated PMA-activated macrophages under the indicated chemotactic conditions. (**F**) Tumor nodes of S100A7-overexpressing and vector-containing control KYSE150 clones mixed with or without activated macrophages (Mφ). (**G**) Subcutaneous xenograft growth curves of the indicated treatment groups. ** p<0.01, *** p<0.001.

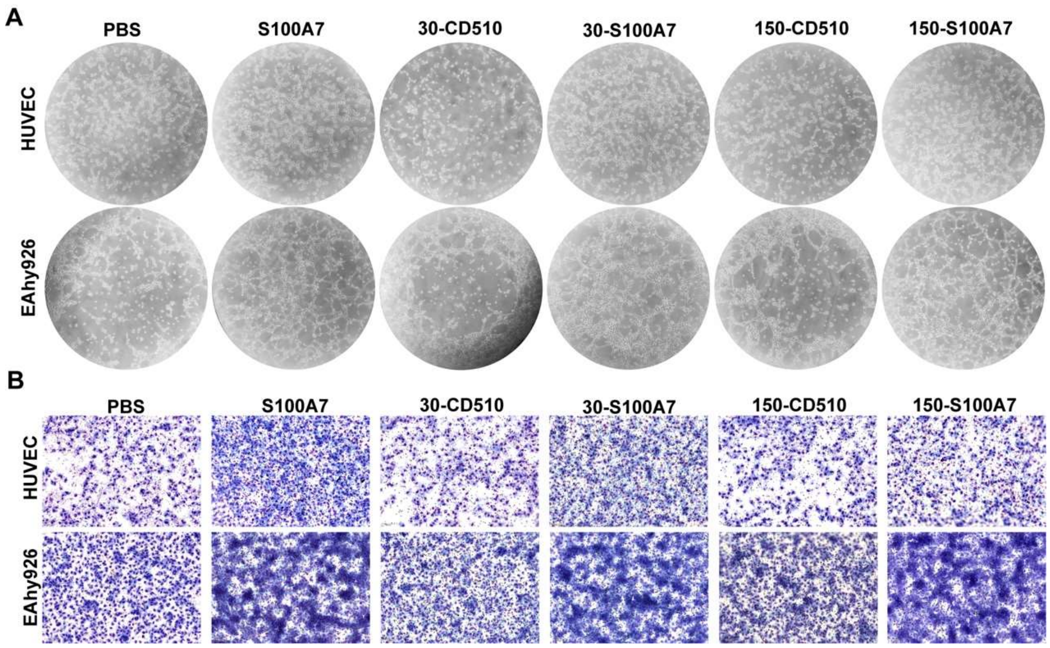

Figure S4. S100A7 promotes tube formation and migration of endothelial cells. (A) Representative pictures of tubules formed by HUVECs and EAhy926 vascular endothelial cells treated with or without S100A7 protein or the indicated culture supernatants. **(B)** Representative pictures of HUVECs and EAhy926 cells passing through chambers treated with or without S100A7 protein or the indicated culture supernatants.

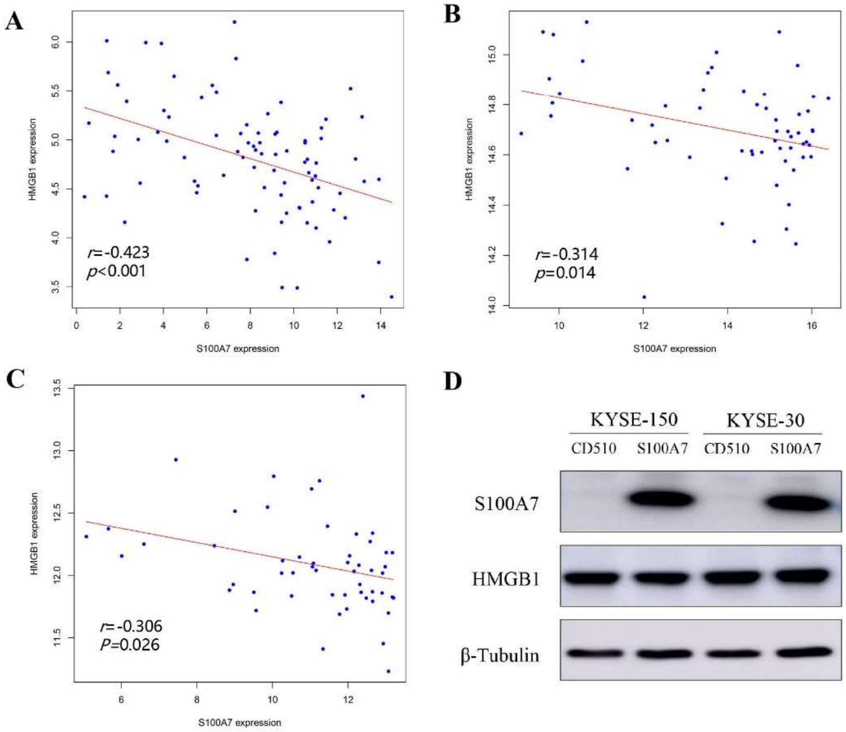

Figure S5. The correlation between S100A7 and HMGB1 in ESCC. (A, B and C) The correlation between S100A7 and HMGB1 by the transcriptome data from TCGA database(A), GSE53622(B) and GSE23400(C). **(D)** Western blot analysis of the correlation between S100A7 and HMGB1 from vector-control and S100A7-overexpressing ESCC cells.

Supplementary Tables

Table S1 Correlation analysis between the expression of free S100A7 in serum and clinicopathological characteristics

Clinicopathological characteristics	S100A7 expression		p value
	High (≥ 1ng/ml)	Low (< 1ng/ml)	
**Gender**			
Man	124 (86.1%)	111 (485.4%)	0.864
Woman	20 (13.9%)	19 (15.6%)	
**Age**			
≤ 60	62 (43.1%)	49 (37.7%)	0.367
> 60	82 (56.9%)	81 (62.3%)	
**Location**			
Upper	26 (18.1%)	27 (20.8%)	0.936
Middle	62 (43.1%)	52 (40.0%)	
Lower	55 (38.2%)	50 (38.5%)	
Unknown	1 (0.7%)	1 (0.8%)	
**Differentiation**			
High grade	25 (17.4%)	15 (11.5%)	0.093
Middle grade	58 (40.3%)	45 (34.6%)	
Low grade	32 (22.2%)	46 (35.4%)	
Unknown	29 (20.1%)	24 (18.5%)	
**T stage**			
T1~T2	32 (22.2%)	44 (33.8%)	0.040
T3~T4	101 (70.1%)	82 (63.1%)	
Unknown	11 (7.6%)	4 (3.1%)	
**Lymphnode metastasis**			
Yes	101 (70.1%)	89 (68.3%)	0.905
No	39 (27.1%)	38 (29.2%)	
Unknown	4 (2.8%)	3 (42.9%)	
**TNM stage**			
I-II	40 (27.8%)	47 (36.2%)	0.243
III-IV	101 (70.1%)	82 (63.1%)	
Unknown	3 (2.1)	1 (0.8%)	

Table S2 Primers used in this study

Genes		Primer Sequences	Length
S100A7	Forward	AACTTCCTTAGTGCCTGTG	19
	Reverse	TGGTAGTCTGTGGCTATGTC	20
GAPDH	Forward	CCTGGTATGACAACGAATTTG	24
	Reverse	CAGTGAGGGTCTCTCTCTTCC	24
CD86	Forward	CCATCAGCTTGTCTGTTTCATTCC	24
	Reverse	GCTGTAATCCAAGGAATGTGGTC	23
CD206	Forward	GGGTTGCTATCACTCTCTATGC	22
	Reverse	TTTCTTGTCTGTTGCCGTAGTT	22
CD163	Forward	TTTGTCAACTTGAGTCCCTTCAC	23
	Reverse	TCCCGCTACACTTGTTTTCAC	21
iNOS	Forward	AGGGACAAGCCTACCCCTC	19
	Reverse	CTCATCTCCCGTCAGTTGGT	20
